# Social contagion models on hypergraphs

**DOI:** 10.1103/PhysRevResearch.2.023032

**Published:** 2020-04-10

**Authors:** Guilherme Ferraz de Arruda, Giovanni Petri, Yamir Moreno

**Affiliations:** ^1^ ISI Foundation, Via Chisola 5, 10126 Torino, Italy; ^2^ Institute for Biocomputation and Physics of Complex Systems (BIFI), University of Zaragoza, 50018 Zaragoza, Spain; ^3^ Department of Theoretical Physics, University of Zaragoza, 50018 Zaragoza, Spain

## Abstract

Our understanding of the dynamics of complex networked systems has increased significantly in the last two decades. However, most of our knowledge is built upon assuming pairwise relations among the system's components. This is often an oversimplification, for instance, in social interactions that occur frequently within groups. To overcome this limitation, here we study the dynamics of social contagion on hypergraphs. We develop an analytical framework and provide numerical results for arbitrary hypergraphs, which we also support with Monte Carlo simulations. Our analyses show that the model has a vast parameter space, with first- and second-order transitions, bistability, and hysteresis. Phenomenologically, we also extend the concept of latent heat to social contexts, which might help understanding oscillatory social behaviors. Our work unfolds the research line of higher-order models and the analytical treatment of hypergraphs, posing new questions and paving the way for modeling dynamical processes on higher-order structures.

Network science has had a radical impact on our knowledge about critical dynamics in complex systems. In particular, new and relevant phenomena arise when investigating social and biological contagion processes [1–7]. For instance, while homogeneous spreading models predict finite critical points [2,6,7], heterogeneous networks often present vanishing transitions [1,2,6–8], supporting the predictions in real-world networks [9–12]. Contagion models cover many aspects, from different types to richer substrates underlying the process itself. A relevant development is the extension of contagion processes to multilayer networks, leading the way to combinatorial higher-order models. Indeed, multilayers' structural [13–17], spreading [7,14], and diffusion properties [13,18] have new and richer phenomenology. Nevertheless, as recently argued in Ref. [19], real data are revealing that pairwise relationships—the fundamental interaction units of networks—do not capture complex dependencies.

Indeed, modern messaging systems (e.g., WhatsApp, Telegram, and Facebook Messenger, among others) allow users to communicate in groups, which creates a direct channel among all members. In other words, modern information spreading is often a one-to-many process. Additionally, biological and team collaborations are also inherently group structured, similarly to some types of molecular interactions [20]. Complementary, there is evidence from social and biological studies indicating that higher-order structures have crucial dynamical effects [21–24]. Therefore, higher-order interactions are ubiquitous, and understanding their properties and impacts is of paramount importance. Notably, the sizes of such groups can span orders of magnitude. Thus, graph-projection-based approaches might not be sufficient to describe systems involving interactions over many different scales and orders.

Combinatorial higher-order models [19] offer a way to describe these systems as they overcome the limitations of lower-order network models. In a first attempt, Bodó *et al.* [21] proposed an SIS disease spreading in a hypergraph. Next, Iacopini *et al.* [22] presented a model of social contagion defined on simplicial complexes and provided approximate solutions for complexes of order three. Their model presented new phenomenological patterns associated with the critical properties of the dynamics. However, their proposed model is still very constrained, both structurally and dynamically. Here we extend their model both structurally and dynamically. Structurally, we adopt hypergraphs, which generalize the concept of graphs, by allowing an edge to have an arbitrary number of nodes (see Fig. 1 for an illustration). Hypergraphs relax the structural restrictions required by simplicial complexes as they impose virtually no limitation on the type, size, and mutual inclusion of interactions, thus, representing more faithfully and naturally real systems. From the dynamical viewpoint, we incorporate explicit critical-mass dynamics (each hyperedge is an independent critical-mass process), generalizing the one modeled in Ref. [22]. The resulting model displays a rich complex phenomenology, remaining very flexible and able to cover a wide range of systems. We uncover the presence of discontinuous transitions and bistability led by higher-order interactions and critical-mass dynamics. Notably, these critical properties contrast with contagion models on graphs-models, which instead usually display continuous transitions, e.g., SIS or SIR disease spreading, regardless of the structural configuration. Therefore, assuming a graph projection of a hypergraph might lead to wrong results. Here we report analytical and numerical analyses of the theoretical framework that we introduce as well as results for several limiting cases and hypergraph structures. We round off the paper by discussing several implications of our study and, most notably, the role of critical mass dynamics in social contagion, providing insights that could help explain reported differences in experimental results [24–27].

**FIG. 1.**
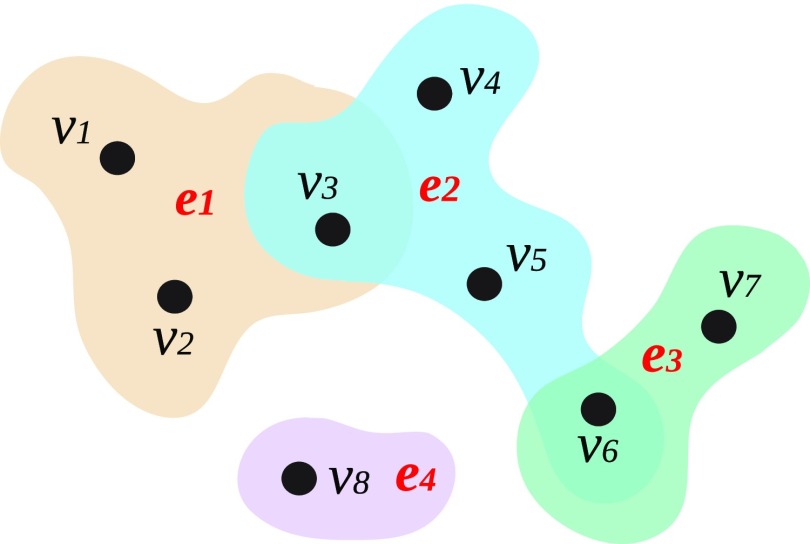
Graphical representation of a hypergraph. Mathematically, $V=\{v_{1},v_{2},v_{3},v_{4},v_{5},v_{6},v_{7},v_{8}\}$, $E=\{e_{1},e_{2},e_{3},e_{4}\}$, where the hyperedges are $e_{1}=\left\{v_{1},v_{2},v_{3}\right\}$, $e_{2}=\left\{v_{3},v_{4},v_{5},v_{6}\right\}$, $e_{3}=\left\{v_{6},v_{7}\right\}$ and $e_{4}=\left\{v_{8}\right\}$.

Let us first introduce some formal definitions. A hypergraph is defined as a set of nodes, $V=\{v_{i}\}$, where N=|V| is the number of nodes, and a set of hyperedges $E=\{e_{j}\}$, where $e_{j}$ is a subset of V with arbitrary cardinality $|e_{j}|$. If $\max\left(|e_{j}|\right)=2$ we recover a graph. We remark that we do not explore this scenario since it recovers standard models. On the other hand, if for each hyperedge with $|e_{j}|>2$ its subsets are also contained in E, we recover a simplicial complex (for more on the hypergraph structure, see the Supplemental Material [28], Sec. I). Figure 1 shows an example of a hypergraph. In an arbitrary hypergraph, we associate with each individual $v_{i}$ a Bernoulli random variable $Y_{i}$ (complementary $X_{i}$). If the node $v_{i}$ is active $Y_{i}=1$ ($X_{i}=0$), otherwise $Y_{i}=0$ ($X_{i}=1$). To each active node, we associate a deactivation mechanism, modeled as a Poisson process with parameter $\delta_{i}$, $N_{i}^{\delta_{i}}$ ($Y_{i}\overset{\delta_{i}}{\to }X_{i}$). For each hyperedge, j, we define a random variable $T_{j}=\sum_{k\in e_{j}}X_{k}$, which is the number of active nodes in the hyperedge. If $T_{j}$ is equal or above a given threshold, $\Theta_{j}$, we associate a Poisson process with parameter $\lambda_{j}$, $N_{j}^{\lambda_{j}}$ (that is, if $T_{j}\geq \Theta_{j}$, then $X_{k}\overset{\lambda_{j}}{\to }Y_{k}$, $\forall k\in e_{j}$). In other words, the dynamics is given by a threshold process that becomes active only above a critical mass of activated nodes. Moreover, if $|e_{j}|=2$, we assume directed Poisson processes, implying that it is not a threshold process anymore. This definition allows for recovering traditional SIS contagion models. While the proposed model is general, allowing for arbitrary heterogeneity in parameters, we focus on more straightforward, but representative, cases. We assume that $\delta_{i}=\delta$ and $\lambda_{j}=f(|e_{j}\left|\right)$, where f is an arbitrary function of the cardinality of the hyperedge. It is also convenient to define $\Theta =\lceil \Theta^{*}N\rceil$, where the parameter $\Theta^{*}$ is a real number representing the fraction of active nodes.

The exact equation that describes the aforementioned dynamics can be written as

(1) $$\begin{array}{c} \frac{dE\left(Y_{i}\right)}{dt}=E\left(-\delta Y_{i}+\left[1-Y_{i}\right]\sum_{e_{j}\cap \left\{v_{i}\right\}\neq \emptyset }\lambda_{j}\sum_{B}1_{\left\{Y_{i}=0,T_{j}\geq \Theta_{j}\right\}}\right), \end{array}$$

where the first summation is over all hyperedges containing $v_{i}$ and the second over all the possible dynamical microstates inside the hyperedge $e_{j}$, denoted by the set B. Furthermore, $1_{\left\{T_{j}\geq \Theta_{j},Y_{i}=0\right\}}$ is an indicator function that is 1 if $Y_{i}=0$ and the critical mass in the hyperedge is reached and 0 otherwise. Naturally, the order parameter is defined as the expected fraction of active nodes, i.e., $\rho =\frac{1}{N}\sum_{i}E\left(Y_{i}\right)$.

Although Eq. (1) captures the exact process, it cannot be numerically solved. Thus, assuming that the random variables are independent and denoting $y_{i}=E\left(Y_{i}\right)$, we obtain the first-order approximation, given as

(2) $$\begin{array}{c} \frac{dy_{i}}{dt}=-\delta y_{i}+\lambda \left(1-y_{i}\right)\sum_{e_{j}\cap \left\{i\right\}\neq \emptyset }\sum_{k=\Theta_{j}}^{|e_{j}|}\lambda^{*}\left(\right|e_{j}\left|\right)P_{e_{j}}\left(K=k\right), \end{array}$$

where we assume that the spreading rate is composed by the product of a free parameter and a function of the cardinality, i.e., $\lambda_{j}=\lambda \times \lambda^{*}\left(\right|e_{j}\left|\right)$. In this formulation, the expectation of the indicator function follows a Poisson binomial distribution (for more on this approximation, see Ref. [28], Sec. II). Formally,

(3) $$E\left(1_{\left\{\left(T_{j}-Y_{k}\right)\geq \Theta_{j}\right\}}\right)\approx \sum_{m=\Theta_{j}}^{|e_{j}|}P_{e_{j}}\left(K=l\right),$$

(4) $$P_{e_{j}}\left(K=l\right)=\sum_{A\in F_{l}}\prod_{i\in A}y_{i}\prod_{j\in A^{c}}\left(1-y_{j}\right),$$

where $F_{l}$ is the set of all subsets of k integers from $\left\{1,2,...,n=|e_{j}|\right\}$, A is one of those sets, and $A^{c}$ is its complementary. Intuitively, A accounts for the possibly active nodes and $A^{c}$ the possibly inactive ones. Thus, the summation over $F_{l}$ considers all possible nodal state configurations in a given hyperedge. Equation (4) is not numerically stable if $|e_{j}|$ is large [29]. Considering the discrete Fourier transform, we obtain a numeric stable solution as [29]

(5) $$P_{e_{j}}\left(K=k\right)=\frac{1}{n+1}\sum_{l=0}^{n}C^{-lk}\prod_{m=1}^{n}\left[1+\left(C^{l}-1\right)y_{m}\right],$$

where $C=\exp\left(\frac{2i\pi }{n+1}\right)$, which then allows one to compute the solution for arbitrarily large hyperedges. Interestingly, although the whole argument is quite intricate, Eq. (5) is simple, allowing the numerical evaluation of Eq. (2).

Our main result is that a rich and diverse phase space, generally populated by continuous and discontinuous transitions and hysteretic behaviors, characterizes contagion on hypergraphs. In particular, we have analytically observed discontinuity and bistability in the order parameter on top of some regular structures. We provide full details of the calculations in Ref. [28] (see Secs. III and IV) for two limiting cases, namely, a hypergraph composed of a hyperedge containing all nodes in addition to (1) a random regular network (which we call a hyperblob) and (2) a star (referred to as a hyperstar).

For the sake of clarity, let us show the main results for the hyperblob. In this case, we can exploit the structural symmetries to solve $\rho (\lambda ,\lambda^{*},\delta )$, obtaining two locally stable solutions. Specifically, consider a hypergraph built up as a homogeneous set of pairwise interactions with average degree 〈k〉 and a single additional hyperedge containing all nodes. In this case, the order parameter can be solved as (for the full derivations, see Ref. [28], Sec. III)

(6) $$\rho^{\text{Lower}}=\left\{\begin{array}{c} 1-\frac{\delta }{\langle k\rangle \lambda },\;\text{if}\;\frac{\lambda }{\delta }\geq \frac{1}{\langle k\rangle }\\ 0,\;\text{otherwise} \end{array}\right.$$

(7) $$\rho^{\text{Upper}}=\frac{-\delta +\langle k\rangle \lambda -\lambda^{*}\lambda +\sqrt{4\langle k\rangle \lambda^{*}\lambda^{2}+\;{\left[\delta +\left(-\langle k\rangle +\lambda^{*}\right)\lambda \right]}^{2}}}{\left(2\langle k\rangle \lambda \right)},$$

where a second-order phase transition for $\rho^{\text{Lower}}$ is naturally obtained as $\frac{\lambda }{\delta }\geq \frac{1}{\langle k\rangle }$ [7]. Furthermore, the discontinuities can also be calculated as

(8) $$\lambda_{c}^{\text{L}}=\frac{\delta }{\langle k\rangle -\Theta^{*}\langle k\rangle },$$

(9) $$\lambda_{c}^{\text{U}}=-\frac{\delta \Theta^{*}}{\lambda^{*}\Theta^{*}-\lambda^{*}+{\left(\Theta^{*}\right)}^{2}\langle k\rangle -\Theta^{*}\langle k\rangle }.$$

Phenomenologically, a discontinuity implies that our system possesses a “social latent heat” that is released or accumulated at a constant value of λ. More specifically, before the discontinuity, “energy” has been stored in the partial activation of the hyperedges. At the discontinuity this “energy” is absorbed (released) at once for a constant value of λ. In fact, the social latent heat can be expressed as

(10) $$Q_{l}\left(\lambda_{c}^{X}\right)={\left[\rho^{\text{Upper}}\left(\lambda ,\delta ,\lambda^{*},N\right)-\rho^{\text{Lower}}\left(\lambda ,\delta ,\lambda^{*},N\right)\right]}_{\lambda =\lambda_{c}^{X}},$$

where $Q_{l}\left(\lambda_{c}^{X}\right)$ can be $Q_{l}\left(\lambda_{c}^{\text{L}}\right)$ (energy absorbed) or $Q_{l}\left(\lambda_{c}^{\text{U}}\right)$ (energy released). Therefore, for this structure, the latent heat is expressed as

(11) $$Q_{l}\left(\lambda_{c}^{X}\right)={\left\{\frac{\delta -\lambda \left(\lambda^{*}+\langle k\rangle \right)+\sqrt{{\left[\delta +\lambda \left(\lambda^{*}-\langle k\rangle \right)\right]}^{2}+4\lambda^{*}\langle k\rangle \lambda^{2}}}{2\langle k\rangle \lambda }\right\}}_{\lambda =\lambda_{c}^{X}},$$

where $\lambda_{c}^{X}$ can be ($\lambda_{c}^{\text{L}}$ or $\lambda_{c}^{\text{U}}$). In fact, this expression is true for any value of λ, but its physical interpretation is valid only near the discontinuity, which in turn depends on $\rho_{c}=\Theta^{*}$. We refer the reader to Ref. [28] for more details.

Figure 2 shows the general phenomenology of the system obtained from the analytical solution, i.e., the first-order approximation, of the equations describing the contagion dynamics for the hyperblob. As can be seen in Fig. 2(a), there are two possible solutions, $\rho^{\text{Lower}}$ and $\rho^{\text{Upper}}$. The solution depends on the initial conditions and the threshold, $\Theta^{*}$, which, together with the structure, defines a value $\rho_{c}$ where the dynamics exhibit a discontinuity. If $\rho \left(t=0\right)\geq \rho_{c}$, the solution is given by $\rho =\rho^{\text{Upper}}$ (forward phase diagram). On the other hand, if $\rho \left(t=0\right)<\rho_{c}$ and ρ(t=0)≠0, then $\rho =\rho^{\text{Lower}}$ (backward phase diagram). The arrows show these solutions as the size of the jump (i.e., the magnitude of the latent heat). Note that the lower solution can exhibit a second-order phase transition. Next, in Fig. 2(b) we instead represent the corresponding parameter space, which is composed of five distinct regions, as explained in the figure caption. We assumed the most general case, where the lower solution has a transition from the absorbing state to an active state, here at $\lambda_{c}$. We remark that, for some structures, the lower solution might have a vanishing critical point (e.g., the hyperstar), i.e., $\lambda_{c}\to 0$, thus slightly changing this picture. Finally, we have compared both analytic and numeric estimates of the latent heat for this hypergraph structure. The results show that the absolute error between analytical and numerical simulations is of order $10^{-2}\text{–}10^{-3}$ in hypergraphs with $N=10^{4}$ (see Ref. [28], Sec. VI.E and Table I), indicating that the first-order approximation is accurate.

**FIG. 2.**
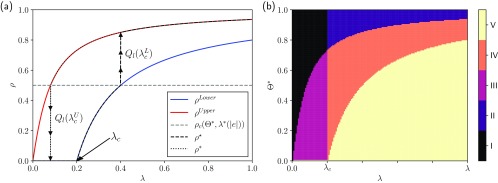
Results for the hyperblob. (a) Possible solutions for a fixed $\Theta^{*}=0.5$. In red and blue are the upper and lower solutions (branches), respectively. The transition from the lower to the upper solution (upper to lower) occurs at the intersection of the lower (upper) solution with a value of $\rho_{c}$ in which the upper solution became stable (unstable). The discontinuity is characterized by the latent heat, $Q_{l}\left(\lambda_{c}^{L}\right)$ or $Q_{l}\left(\lambda_{c}^{U}\right)$. At $\lambda_{c}=0.2$, the lower solution shows a second-order phase transition. (b) Schematic of the parameter space: Region I: the absorbing state for both the lower and upper solution; Region II: only the lower solution is stable (the global critical mass is not reached, $\rho <\rho_{c}$); Region III: $\rho^{\mathrm{Upper}}$ is stable and $\rho^{\text{Lower}}=0$ (below the critical point); Region IV: $\rho^{\mathrm{Upper}}>\rho^{\mathrm{Lower}}>0$ and both are stable (bistable); Region V: only the upper solution is stable (the global critical mass was reached, $\rho \geq \rho_{c}$).

Thus, generically, the solutions for a social contagion dynamics on hypergraphs can be mathematically expressed as

(12) $$\begin{array}{ccc} & \rho^{★}= & \left\{\begin{array}{c} \rho^{\text{Lower}}\;\text{if}\;\rho^{\text{Lower}}<\rho_{c}\\ \rho^{\text{Upper}}\;\text{if}\;\rho^{\text{Lower}}\geq \rho_{c} \end{array}\right., \end{array}$$

(13) $$\begin{array}{ccc} & \rho^{*}= & \left\{\begin{array}{c} \rho^{\text{Upper}}\;\text{if}\;\rho^{\text{Upper}}\geq \rho_{c}\\ \rho^{\text{Lower}}\;\text{if}\;\rho^{\text{Upper}}<\rho_{c} \end{array}\right., \end{array}$$

where $\rho^{★}$ is obtained if $\rho \left(t=0\right)<\rho_{c}$, and $\rho^{*}$ if $\rho \left(t=0\right)\geq \rho_{c}$, where $\rho_{c}$ is a global critical mass, i.e., the value of ρ at which the discontinuity appears. As before, we note that the lower solution (branch) might also exhibit a second-order (continuous) phase transition, denoted by $\lambda_{c}$ in Fig. 2, from the absorbing state (ρ=0) to the active state (ρ>0). Furthermore, for a given hypergraph with fixed δ and $\lambda^{*}$, the discontinuity points are formally defined as

(14) $$\begin{array}{ccc} & \lambda_{c}^{\text{L}}= & {\text{arg}}_{\lambda }\left[\rho^{\text{Lower}}\left(\lambda ,\delta ,\lambda^{*},N\right)=\rho_{c}\right], \end{array}$$

(15) $$\begin{array}{ccc} & \lambda_{c}^{\text{U}}= & {\text{arg}}_{\lambda }\left[\rho^{\text{Upper}}\left(\lambda ,\delta ,\lambda^{*},N\right)=\rho_{c}\right], \end{array}$$

thus, also defining the bistable region, $\left(\lambda_{c}^{\text{U}},\lambda_{c}^{\text{L}}\right)$.

Although a closed solution for the general case is not possible, Monte Carlo simulations and numerical evaluation of Eq. (2) are reasonable alternatives to characterize our system (see Ref. [28], Sec. VI). Here we focus on a hypergraph with an exponential distribution of cardinalities (i.e., the number of nodes inside a hyperedge), $P\left(|e|\right)∼\mu \exp\left(-\mu |e|\right)$ with the constraint that |e|≥2. Dynamically, we set $\lambda^{*}=\log_{2}\left(|e|\right)$. This choice is arbitrary, but we choose here the $\log_{2}\left(|e|\right)$ function because it grows sublinearly. Note that, if a hyperedge cardinality goes to infinity, the average spreading value tends to zero: $\lim_{|e|\to \infty }\frac{\log_{2}\left(|e|\right)}{\left|e\right|}$ = 0. The impact of such a function is yet unknown, and we leave this analysis for future work.

Figures 3(a) and 3(b) show that the order parameter and the susceptibility follow the patterns expected for a first-order transition, i.e., both are discontinuous. Moreover, the order parameter is bistable, implying the presence of a hysteresis loop. This phenomenon is opposed to an SIS on a graph. The SIS has a second-order phase transition, characterized by a continuous behavior of the order parameter and a diverging susceptibility in the thermodynamic limit. Complementarily, Fig. 3(c) shows the distribution of active nodes in the upper and lower branches. In the former, we have a bell-shaped distribution, similar to the supercritical regime of an SIS process [7,30]. In the latter, we have a distribution peaked at one, similar to the subcritical regime (absorbing state) of an SIS process [7,30]. We emphasize that Fig. 3(c) displays the distribution of active nodes for the upper (left panel) and lower (right panel) branches and that the complete distribution for a given λ in a region where both solutions exist is bimodal. Intuitively, one expects that lower cardinality hyperedges are responsible for the lower branch as they are easier to activate than the higher cardinality ones. Note that, in regular cases, such as the hyperblob (see Ref. [28], Secs. III and IV), the giant pairwise component has N nodes. However, for the exponentially distributed cardinality scenario, this is not the case. Although $P\left(|e|=2\right)\geq P\left(|e|=k\right)$, k=3,4...,N (exponential distribution), the largest connected component, is very small, six nodes in the simulated hypergraph. Therefore, in this scenario, the lower branch is determined by hyperedges with cardinality lower but greater than two. The generality of the reported phenomenological behavior suggests that the observed dynamics are a consequence of group-group interactions. Additional experiments (reported in Ref. [28]) for a hypergraph with a power-law distribution of cardinality further corroborated these results. In all systems, we found similar qualitative behavior for finite networks.

**FIG. 3.**
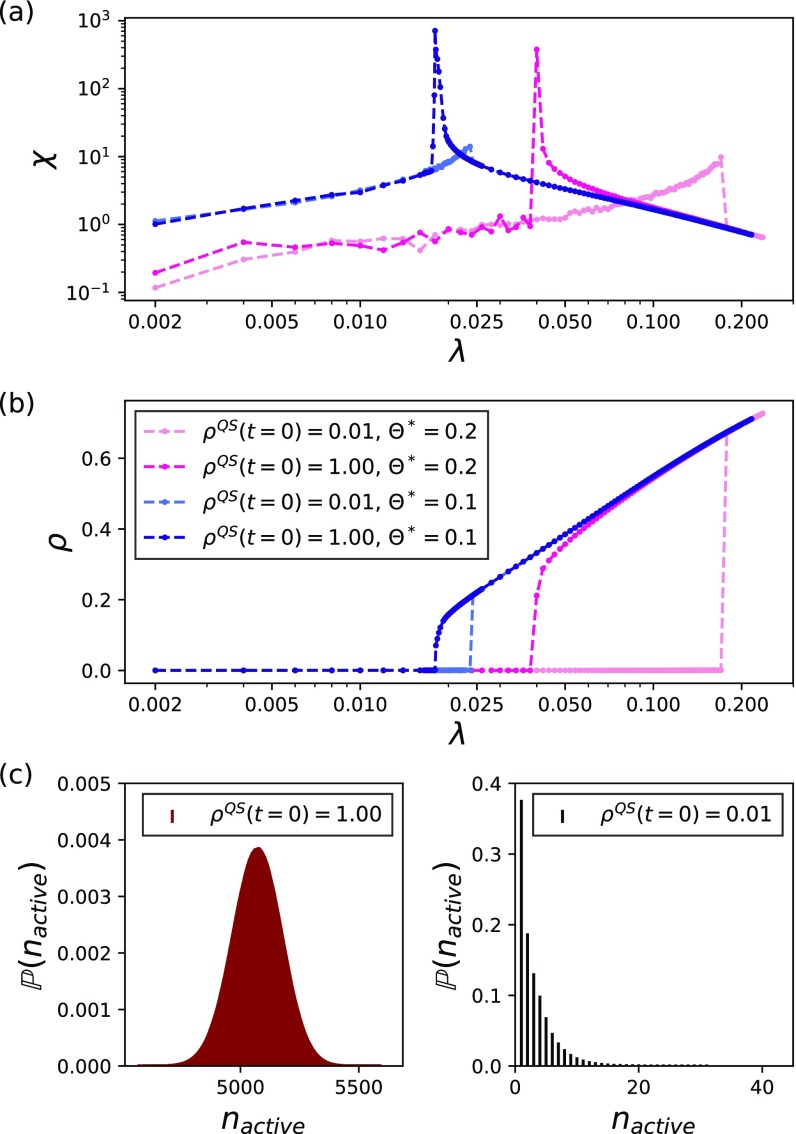
Estimation of ρ and χ using the QS method in a hypergraph with an exponential distribution of hyperedge cardinalities and $N=10^{4}$. The dynamical parameter are: δ=1.0, $\lambda^{*}=\log_{2}\left(\right|e_{j}\left|\right)$, and $\Theta^{*}=0.1,0.2$. (a) The susceptibility; (b) the order parameter. We considered two initial conditions for the QS method, $\rho^{QS}\left(t=0\right)=0.01$, darker colors, and $\rho^{QS}\left(t=0\right)=1.00$ lighter colors. (c) The distribution of active node estimated using the QS method at λ=0.086 and $\Theta^{*}=0.2$ [the crossing between the two susceptibility curves, in Fig. 3(a)].

Our results are relevant because they provide a theoretical foundation for, and a phenomenological explanation to, seemingly different experimental findings [24–27,31–33]. These works reported critical mass levels needed to change an established equilibrium of 10% in some experiments and 30%–40% in others, in apparent contradiction. The formalism here developed naturally brings forth plausible hypotheses for these observations and show that both ranges are possible. On the one hand, studies based on a single group suggest a threshold between 30%–40%, a situation that can be modeled as a single hyperedge in our formalism. On the other hand, a critical mass of 10% would correspond to a population that is composed of groups of diverse sizes, each one with a (larger) activation threshold. In other words, it is possible to have individual groups exhibiting a threshold $\Theta^{*}$ between 30%–40%, and at the same time, a global critical mass, $\rho_{c}$, for the whole population of about 10% due to group intersections. A second reason that could explain the experimental findings is even more straightforward: admittedly, the fact that our model shows bistability also enables, for a given λ, two possible solutions for ρ corresponding to the lower and the upper branches. That is, the system might be operating in the region where both solutions are larger than zero and stable.

In summary, in this paper, we have developed a framework that allows extending the study of social contagion models when group interactions are relevant. This is achieved by considering hypergraphs as the substrates that capture such many-to-many interactions. First, our work opens the path to deal with new dynamical processes on top of higher-order models and specifically on hypergraphs. Second, we showed that simple dynamical processes could exhibit very rich dynamics, with different transitions, bistability, and hysteresis. Hypergraphs are ubiquitous, and our theory suggests that such a structure allows for the phase diagram reported here. Several findings support the relevance of this methodology. We remark that, depending on the structure, traditional graph-projected models may lead to wrong results. Ultimately, the uncovered phenomenology allows explaining seemingly contradictory experimental findings in which group interactions play a major role. We also mention that many interesting questions arise from our work. For instance, if one assumes that energy is proportional to ρ, our model might display phenomena reminiscent of a Carnot cycle for social contexts, which might help to understand abrupt changes and oscillatory patterns in social behaviors.
